# Statistical Downscaling of General Circulation Model Outputs to Precipitation Accounting for Non-Stationarities in Predictor-Predictand Relationships

**DOI:** 10.1371/journal.pone.0168701

**Published:** 2016-12-20

**Authors:** D. A. Sachindra, B. J. C. Perera

**Affiliations:** Institute for Sustainability and Innovation, College of Engineering and Science Victoria University, Melbourne, Victoria, Australia; Universidade de Vigo, SPAIN

## Abstract

This paper presents a novel approach to incorporate the non-stationarities characterised in the GCM outputs, into the Predictor-Predictand Relationships (PPRs) in statistical downscaling models. In this approach, a series of 42 PPRs based on multi-linear regression (MLR) technique were determined for each calendar month using a 20-year moving window moved at a 1-year time step on the predictor data obtained from the NCEP/NCAR reanalysis data archive and observations of precipitation at 3 stations located in Victoria, Australia, for the period 1950–2010. Then the relationships between the constants and coefficients in the PPRs and the statistics of reanalysis data of predictors were determined for the period 1950–2010, for each calendar month. Thereafter, using these relationships with the statistics of the past data of HadCM3 GCM pertaining to the predictors, new PPRs were derived for the periods 1950–69, 1970–89 and 1990–99 for each station. This process yielded a non-stationary downscaling model consisting of a PPR per calendar month for each of the above three periods for each station. The non-stationarities in the climate are characterised by the long-term changes in the statistics of the climate variables and above process enabled relating the non-stationarities in the climate to the PPRs. These new PPRs were then used with the past data of HadCM3, to reproduce the observed precipitation. It was found that the non-stationary MLR based downscaling model was able to produce more accurate simulations of observed precipitation more often than conventional stationary downscaling models developed with MLR and Genetic Programming (GP).

## Introduction

General Circulation Models (GCMs) are the main tools used for projection of global climate into the future [1], and they are based on the mathematical representations of physics of the atmosphere, ocean, ice caps and land surface processes [2]. For projecting the global climate into the future, GCMs are forced with various scenarios of future greenhouse gas (GHG) emissions. GCMs can reliably simulate the global climate at a coarse spatial resolution in the order of few hundred kilometres. However, owing to their coarse spatial resolution they cannot adequately simulate the catchment scale climate, which is largely influenced by the subgrid-scale features such as topography, land use, and convective processes [3]. The majority of the catchment scale applications need hydroclimatic data at a much finer spatial resolution than that of GCM outputs [4]. Therefore, to bridge the spatial scale gap between the coarse resolution GCM outputs and the fine resolution hydroclimatic information required for catchment scale applications, dynamic and statistical downscaling approaches are used [5].

In dynamic downscaling, GCM outputs are fed into a Regional Climate Model (RCM) as lateral boundary conditions [6]. The high computational cost is a major limitation in dynamic downscaling. Statistical downscaling is based on the concept of developing empirical statistical relationships between the GCM outputs and the catchment scale hydroclimatic variables [7]. Statistical downscaling depends on the assumption that the relationships between the GCM outputs (predictors—inputs to downscaling models) and the observations of the catchment scale hydroclimatic variables (predictands—outputs of downscaling models) in the past are valid for the future under changing climate [8]. This assumption is called the stationarity assumption of the predictor-predictand relationships (PPRs). Unlike dynamic downscaling, statistical downscaling is associated with much less computational costs [9] and hence it has gained wide popularity in the downscaling scientific community [10].

The projections of hydroclimatic variables produced in statistical downscaling studies are subject to uncertainties that arise from numerous sources (some of these uncertainties are also valid for dynamic downscaling). The sources of uncertainties in a downscaling study include: GHG emission scenarios used to force GCMs, GCMs used to provide inputs to downscaling models, various techniques used for developing downscaling models, non-homogeneity in inputs to downscaling models, specific methodologies employed in developing downscaling models (e.g. how predictors are selected and pre-processed, use of cross-validation instead of traditional calibration and validation, and how outputs are post-processed), stationarity assumption of PPRs and stationarity assumption of bias/bias-correction approaches (bias refers to errors in downscaling model outputs), and the quality of observations against which downscaling models are calibrated [11]. These uncertainties can largely influence the future hydroclimatic projections.

To the date some of the above sources of uncertainties have been investigated in detail and in certain instances potential solutions to reduce the uncertainties that arise from those sources have been developed. As examples: use of multiple GHG emission scenarios to quantify the impact of changes in the anthropogenic GHG emissions on catchment scale climate [12, 13, 14], scenario neutral approach for reducing the dependence on multiple GHG emission scenarios [15], identification of GCMs that better simulate the climate over the region of interest [16], use of ensemble techniques to combine the outputs of downscaling models produced with outputs of different GCMs [17], and derivation of homogeneous inputs to downscaling models [18]. In statistical downscaling the uncertainties arising from the stationarity assumption of PPRs and stationarity assumption of bias/bias-correction approaches, have not been investigated adequately. However, the validity of the stationarity assumption of the PPRs is questionable under non-stationary climate likely to occur in the future [19].

It has been found that the constants and coefficients in the PPRs developed using multi-linear regression (MLR) technique can be used to detect non-stationarities in the PPRs [20, 21]. However, in these studies a methodology to account for the non-stationarities in the PPRs of statistical downscaling models under changing climate was not investigated. If non-stationarities in the PPRs are observed in the past, there is a high likelihood that such non-stationarities in the PPRs will be present in the future, under non-stationary climate [20]. Therefore, there is a need for developing downscaling approaches that can explicitly account for the non-stationarities in the PPRs under non-stationary climate [11]. To the date, there are only a few studies that have investigated potential approaches for handling non-stationarities in the PPRs in statistical downscaling [22]. In these studies, potential approaches for handling non-stationarities in the PPRs in statistical downscaling have been developed: considering the changes in the occurrence frequency of modes of natural variability of climate as an indicator of changes in the climate [23, 19], using a moving window approach [22] and employing wavelet transformation [24].

This paper presents a novel approach based on the use of a moving window to incorporate the non-stationarities in the large scale climate in the PPRs in statistical downscaling models. The next sections of the paper provide the details of “Study Area and Data”, “Methodology”, “Results” and “Discussion” in relation to a case study.

## Study Area and Data

**Fig 1:**
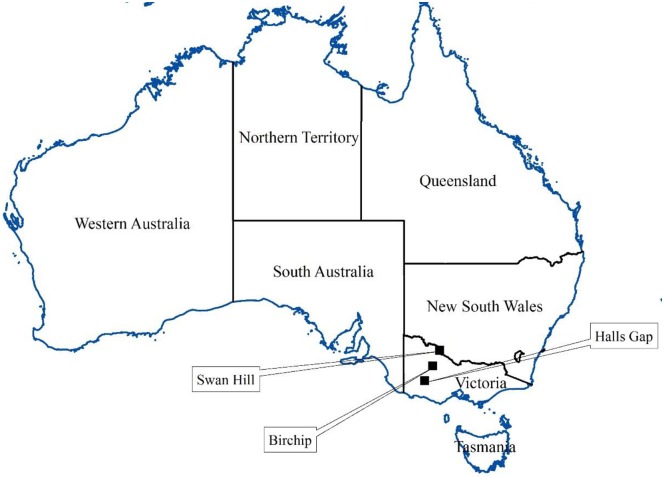
Locations of precipitation stations.

The operational area (about 62,000 km^2^) of Grampians Wimmera Mallee Water Corporation (GWMWater) is located in the northwestern part of Victoria, Australia. For the demonstration of the methodology, precipitation stations at Halls Gap (Lat. −37.14°, Lon. 142.52°, elevation 257 m from mean sea level), Birchip (Lat. −35.98°, Lon. 142.92°, elevation 102 m from mean sea level) and Swan Hill (Lat. −35.34°, Lon. 143.55°, elevation 70 m from mean sea level) in the operational area of GWMWater were selected. This study area was selected purely for the demonstration of the methodology detailed in the paper. The annual averages of precipitation at Halls Gap, Birchip and Swan Hill are about 950 mm, 375 mm and 360 mm respectively. These 3 stations were selected in such way that they represent relatively wet, intermediate and relatively dry climate regimes in the study area. The selection of stations representative of relatively wet, intermediate and relatively dry climate regimes enabled the testing of the robustness of the downscaling approaches. The locations of the precipitation stations at Halls Gap, Birchip and Swan Hill are shown in Fig 1.

For the calibration and validation (calibration and validation together refer to model development) of the statistical downscaling models, observations of the predictand and reanalysis data pertaining to the predictors are required. For this investigation, daily observed precipitation for the observation stations at Halls Gap, Birchip and Swan Hill were obtained from the SILO database (http://www.longpaddock.qld.gov.au/silo/) of the Queensland Climate Change Centre of Excellence for the period 1950–2010. In the SILO database for infilling the missing daily precipitation values, ordinary kriging method has been used to spatially and temporally interpolate daily rainfall [25]. In order to analyze the temporal and spatial errors in the interpolated precipitation data, independent cross validation technique has been used. These daily observations were then added to produce monthly precipitation totals for the development of downscaling models. In general, water resources allocation modelling is conducted at a monthly time step and hence it needs monthly hydroclimatic inputs. Therefore, the scope of this study was limited to the investigation of downscaling models operated at a monthly time step.

The monthly reanalysis data pertaining to predictors were obtained for the period 1950–2010 from the National Oceanic and Atmospheric Administration / Earth System Research Laboratory (NOAA/ESRL) Physical Sciences Division. The outputs of HadCM3 (a GCM) pertaining to the 20^th^ century climate experiment (20C3M) were obtained from the Programme for Climate Model Diagnosis and Inter-comparison (http://www-pcmdi.llnl.gov/) for the period 1950–1999. The monthly 20C3M outputs of HadCM3 were used for reproducing the past observed precipitation as it allows the assessment of the ability of the downscaling models (developed with reanalysis outputs) in reproducing the observed precipitation with GCM outputs. HadCM3 is one of the GCMs that can properly simulate the precipitation over Australia and the El Niño Southern Oscillation [16]. Therefore, HadCM3 was used to provide the inputs to the downscaling models.

## Methodology

### Overview of methodology

In the non-stationary downscaling approach, initially, a series of PPRs (i.e. downscaling models) were determined with the MLR technique by using a moving window on the past predictor data obtained from the NCEP/NCAR reanalysis data archive and observations of precipitation at each station. Then the relationships between the constants/coefficients in these PPRs and the statistics of past reanalysis data of predictors were determined. The non-stationarities in the climate are characterised by the variations in the statistics of the climate variables over time. Hence, the above process enabled linking the non-stationarities in the climate to the PPRs. These PPRs developed using reanalysis data were then modified according to the statistics of predictor data pertaining to the past climate simulated by the 20C3M outputs of HadCM3, yielding new PPRs corresponding to the past climate characterised by HadCM3. The ability of these PPRs to reproduce the past catchment scale precipitation with the outputs of a GCM was considered as an indication of the success of the non-stationary modelling approach. The series of PPRs developed using the above moving window approach resembles a non-stationary downscaling model and it is denoted as SDM_1(MLR)_.

**Fig 2:**
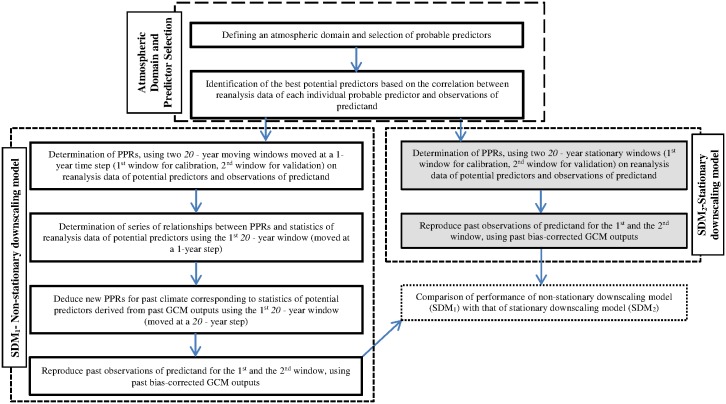
Steps involved in the stationary and non-stationary downscaling approaches.

The performance of this non-stationary modelling approach was compared with that of a stationary modelling approach. Under the stationary modelling approach, linear and non-linear downscaling models were developed using MLR and Genetic Programming (GP). These stationary downscaling models developed with MLR and GP are denoted as SDM_2(MLR)_ and SDM_2(GP)_, respectively. The stationary downscaling models SDM_2(MLR)_ and SDM_2(GP)_ are denoted as SDM_2_ in general. The flow chart in Fig 2 shows the steps involved in the development of non-stationary and stationary downscaling models. It should be noted that the steps shown in Fig 2 were applied to each precipitation station separately.

The next sub-sections provide the details of the steps shown in Fig 2 in the following order under titles: Defining an atmospheric domain and selection of predictors, Development of non-stationary downscaling models (SDM_1_) (*which includes the sub-sections*: *Relationships between PPRs in SDM*_*1*_ *and statistics of reanalysis data*, *Modification of PPRs in SDM*_*1*_ *according to statistics of GCM outputs*, *Bias correction of GCM outputs and reproduction of observed precipitation*) and Development of stationary downscaling models (SDM_2_).

### Defining an atmospheric domain and selection of predictors

**Fig 3:**
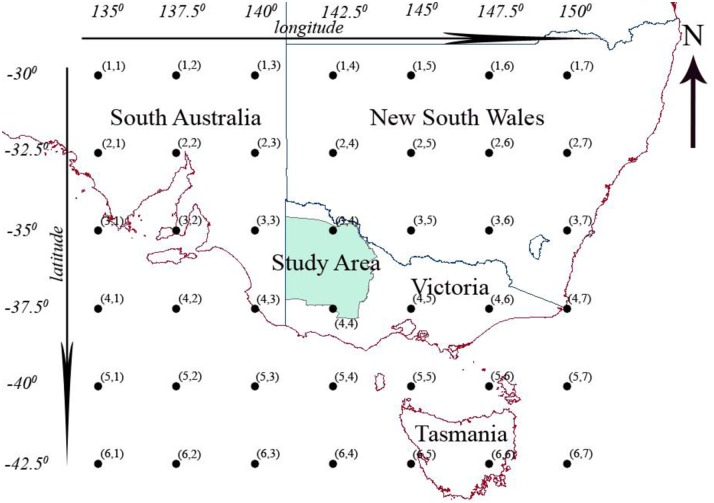
Atmospheric domain for downscaling.

The atmospheric domain in a statistical downscaling study is the region of the atmosphere corresponding to which the large scale atmospheric information is obtained, for providing inputs to a downscaling model. In this investigation an atmospheric domain that covered the region bounded by the longitudes 135°E—150°E and latitudes 30°S—42.5°S was selected, and it is shown in Fig 3. It should be noted that for all downscaling models the same atmospheric domain was used.

In statistical downscaling, it is the common practice to select an initial pool of predictors called probable predictors that are the most likely to influence the predictand of interest. The set of probable predictors selected for this study consisted of; 500 hPa, 700 hPa, 850 hPa and 1000 hPa relative humidity; 500 hPa, 700 hPa, 850 hPa and 1000 hPa specific humidity; 500 hPa, 700 hPa, 850 hPa, 1000 hPa air temperature; surface air temperature; 200 hPa, 500 hPa, 700 hPa, 850 hPa and 1000 hPa geopotential heights; mean sea level pressure; surface pressure; surface precipitation rate; 850 hPa zonal wind speed; and 850 hPa meridional wind speed. These probable predictors were selected based on the previous downscaling studies over the same study area [17, 26, 27].

Potential predictors are the most influential predictors on the predictand of interest selected from the pool of probable predictors. The potential predictors vary spatiotemporally as the relationships between the large-scale atmospheric conditions and the catchment scale climate can vary over time and space [24]. In order to identify the potential predictors, initially, for each calendar month, the NCEP/NCAR reanalysis data of probable predictors pertaining to the 42 grid points (shown in Fig 3) and observed precipitation data at Halls Gap, Birchip and Swan Hill for the period 1950–2010 were split into three 20-year time slices in the chronological order. Thereafter, the Pearson correlations coefficients between the probable predictors and precipitation were computed for each time slice and the whole period (period which contained all 20-year time slices) for each grid point in the atmospheric domain. Then the probable predictors which displayed statistically significant correlations at the 95% confidence level with observed precipitation with a consistent sign (no changes in the sign of the correlation over time) in all three time slices and the whole period were selected as potential predictors for each calendar month for each station. For each calendar month the three potential predictors that showed the highest correlations with observed precipitation were selected as inputs to both stationary and non-stationary downscaling models. In the selection of the three best potential predictors, when a predictor showed statistically significant high correlations corresponding to multiple grid locations of the atmospheric domain, only the best correlated location was considered.

Above predictor selection procedure assisted in avoiding the influx of any redundant information to the downscaling models. These three potential predictors selected for each calendar month are called the best potential predictors. In Table 1 the three best potential predictors identified for each calendar month are shown in the descending order of the magnitude of the correlation coefficients which they showed with the observations of precipitation at Halls Gap, Birchip and Swan Hill over the period 1950–2010. As shown in Fig 2 defining an atmospheric domain and selection of predictors was a common step for both downscaling approaches.

**Table 1:** Three best potential predictors identified for each calendar month for each station.

Station	Month	Three best potential predictors used for SDM_1_ and SDM_2_ with grid locations
Halls Gap	January	surface precipitation rate {(4,4)}, 1000 hPa^a^ specific humidity {(3,3)}, 850 hPa meridional wind {(6,3)}
	February	surface precipitation rate {(4,3)}, 1000 hPa relative humidity {(4,3)}, 850 hPa relative humidity {(3,1)}
	March	surface precipitation rate {(3,4)}, 850 hPa relative humidity {(2,3)}, 1000 hPa specific humidity {(4,4)}
	April	850 hPa relative humidity {(4,4)}, 850 hPa geopotential height {(1,3)}, surface precipitation rate {(2,4)}
	May	surface precipitation rate {(4,4)}, mean sea level pressure {(4,4)}, 1000 hPa geopotential height {(5,4)}
	June	surface precipitation rate {(3,4)}, mean sea level pressure {(3,5)}, 850 hPa zonal wind {(4,2)}
	July	850 hPa zonal wind {(4,1)}, 850 hPa geopotential height {(3,4)}, mean sea level pressure {(4,4)}
	August	surface precipitation rate {(3,4)}, 850 hPa geopotential height {(4,6)}, mean sea level pressure {(5,6)}
	September	surface precipitation rate {(3,4)}, 850 hPa relative humidity {(3,3)}, 700 hPa relative humidity {(3,3)}
	October	surface precipitation rate {(3,4)}, 850 hPa relative humidity {(3,4)}, 700 hPa geopotential height {(2,2)}
	November	850 hPa relative humidity {(3,3)}, surface precipitation rate {(3,4)}, 1000 hPa relative humidity {(3,3)}
	December	surface precipitation rate {(4,4)}, 850 hPa relative humidity {(2,3)}, 850 hPa specific humidity {(6,6)},
Swan Hill	January	1000 hPa specific humidity {(3,3)}, surface precipitation rate {(3,3)}, 850 hPa specific humidity {(3,2)}
	February	surface precipitation rate {(5,4)}, 1000 hPa specific humidity {(4,3)}, 700 hPa relative humidity {(5,3)}
	March	surface precipitation rate {(4,3)}, 1000 hPa specific humidity {(4,3)}, 850 hPa specific humidity {(6,4)}
	April	1000 hPa specific humidity {(2,3)}, surface precipitation rate {(3,2)}, 850 hPa specific humidity {(4,4)}
	May	surface precipitation rate {(5,4)}, 700 hPa relative humidity {(6,3)}, 1000 hPa relative humidity {(6,3)}
	June	surface precipitation rate {(1,2)}, 700 hPa relative humidity {(5,3)}, 850 hPa specific humidity {(1,1)}
	July	surface precipitation rate {(3,3)}, 1000 hPa specific humidity {(5,3)}, 700 hPa relative specific humidity {(3,1)}
	August	surface precipitation rate {(5,3)}, 700 hPa relative humidity {(4,2)}, 1000 hPa relative humidity {(2,3)}
	September	surface precipitation rate {(3,2)}, 700 hPa relative humidity {(2,3)}, 500 hPa specific humidity {(4,2)}
	October	surface precipitation rate {(5,3)}, 850 hPa relative humidity {(6,3)}, 1000 hPa relative humidity {(6,3)}
	November	surface precipitation rate {(4,3)}, 1000 hPa relative humidity {(5,3)}, 1000 hPa specific humidity {(5,4)}
	December	1000 hPa specific humidity {(3,2)}, surface precipitation rate {(3,1)}, 1000 hPa relative humidity {(3,1)}
Birchip	January	surface precipitation rate {(3,2)}, 1000 hPa specific humidity {(3,3)}, 850 hPa relative humidity {(2,1)}
	February	surface precipitation rate {(4,4)}, 850 hPa relative humidity {(6,2)}, 700 hPa relative humidity {(6,1)}
	March	surface precipitation rate {(5,4)}, 1000 hPa specific humidity {(6,4)}, 850 hPa specific humidity {(2,3)}
	April	850 hPa specific humidity {(3,3)}, 700 hPa relative humidity {(4,3)}, 1000 hPa specific humidity {(2,3)}
	May	surface precipitation rate {(2,3)}, 700 hPa relative humidity {(4,1)}, 1000 hPa relative humidity {(6,3)}
	June	surface precipitation rate {(3,4)}, 700 hPa relative humidity {(3,2)}, 1000 hPa specific humidity {(5,3)}
	July	surface precipitation rate {(5,4)}, 850 hPa specific humidity {(3,2)}, 1000 hPa specific humidity {(5,3)}
	August	surface precipitation rate {(5,3)}, 700 hPa relative humidity {(1,2)}, 850 hPa relative humidity {(7,6)}
	September	surface precipitation rate {(5,4)}, 1000 hPa specific humidity {(5,4)}, 700 hPa relative specific humidity {(2,3)}
	October	surface precipitation rate {(3,3)}, 850 hPa relative humidity {(1,3)}, 1000 hPa relative humidity {(5,3)}
	November	surface precipitation rate {(5,4)}, 1000 hPa relative humidity {(2,1)}, 850 hPa relative humidity {(2,2)}
	December	surface precipitation rate {(4,4)}, 1000 hPa specific humidity {(5,4)}, 1000 hPa relative humidity {(6,3)}

^a^atmospheric pressure in hectopascal

### Development of non-stationary downscaling models (SDM_1_)

For the development of SDM_1(MLR)_, for each calendar month, for each station, two 20-year moving windows were defined in such a way that the 1^st^ and the 2^nd^ moving windows initially covered the 1^st^ and the 2^nd^ 20-years of the reanalysis data of the three best potential predictors (refer to Table 1) and the observations of precipitation over the period 1950–2010. Then for each calendar month, these two windows were moved at a 1-year time step. The 1^st^ window was moved until it swept over the whole set of data (1950–2010), and for that, 41 1-year movements were required. Once the 1^st^ window was moved to the position where it covered the data of the period 1971–1990, the 2^nd^ window would have covered the last 20-years of data which corresponded to the period 1991–2010. Thereafter, for each movement of the two windows, data were concatenated for the 2^nd^ window by considering data from 1950 onwards. (e.g. in determining the PPRs for period 1972–1991, the 2^nd^ window would have covered period 1992–2010 and year 1950).

For each 1-year movement of the two windows and also for their original positions, the reanalysis data pertaining to the three best potential predictors were standardised using their means and the standard deviations. Then using these standardised data, the constant and the coefficients of PPRs in SDM_1(MLR)_ were determined for each movement of the 1^st^ moving window by minimising the sum of squared errors between the model-reproduced values and observed precipitation. This phase of the model development is called model calibration. The PPRs of SDM_1(MLR)_ had the format given in Eq 1, where *Y* is the predictand and *b*_*i*_ is the coefficient of predictor *x*_i_, D is the constant and ε is the Gaussian error. Since *i* = 3 there are three coefficients *b*_*1*_, *b*_*2*_ and *b*_*3*_ corresponding to the three best potential predictors *x*_1_, *x*_2_ and *x*_3_ respectively.

$$Y=\text{D}+\;\sum_{i=1}^{3}\left(b_{i}x_{i}\right)\;+\;\varepsilon$$ (1)

Then the PPRs developed for the 1^st^ moving window were used to reproduce the observed precipitation pertaining to the 2^nd^ moving window as a validation of the performance of SDM_1(MLR)_. The calibration and validation process yielded a series of 42 PPRs for each calendar month for each station (total number of PPRs = 12 x 42) corresponding to the 1^st^ moving window. The use of moving windows at a 1-year step enabled the identification of fine changes in the PPRs even with a short record of data.

**Fig 4:**
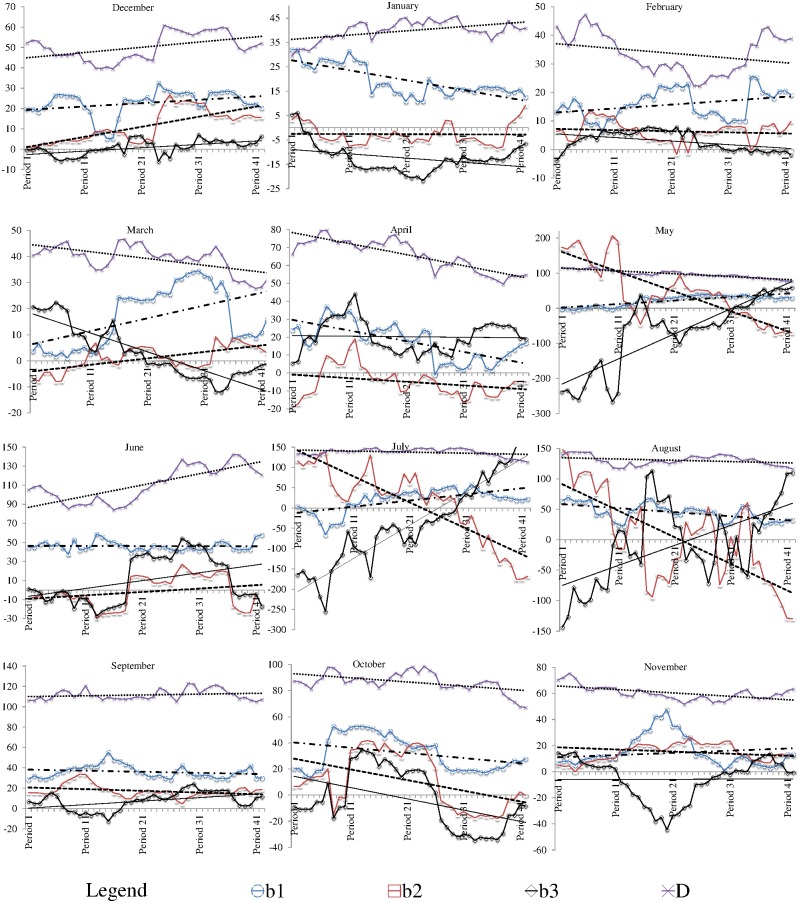
Time series of coefficients and constant in MLR based PPRs for Halls Gap for summer, autumn, winter and spring (Period 1 = 1950–69, Period 2 = 1951–70……Period 42 = 1991–2010).

The time series of the coefficients and the constant in the PPRs of SDM_1(MLR)_ are shown in Fig 4 for the precipitation station at Halls Gap. In Fig 4, each period (horizontal axis) refers to a 20-year time slice defined by the movement of the 1^st^ window (i.e. Period 1 = 1950–69, Period 2 = 1951–70……Period 42 = 1991–2010). According to Fig 4 it was seen that in all calendar months the constant in the PPRs showed either a distinct increasing or a decreasing trend over time. Also, the three coefficients *b*_*1*_, *b*_*2*_ and *b*_*3*_ in the majority of the calendar months displayed either an increasing or a decreasing trend over time, with significant fluctuations in certain calendar months (e.g. October).

A test was then conducted to determine the statistical significance of the linear trends [28] of each coefficient and the constant in the PPRs for each calendar month for each station. The results of the statistical significance test are shown in Table 2. According to Table 2, it was realised that, the linear trends in the coefficients and constants in the PPRs were significant at the 95% confidence level in the majority of the calendar months at all stations. These significant trends in the coefficients and constants in the PPRs are indicative of the influence of non-stationarities in the large scale climate on the catchment scale precipitation. However, it should be noted that the changes in the constant and the coefficients of the series of PPRs could be due to other factors such as natural variability of the climate and data inconsistencies. The identification of such influences on the PPRs is beyond the scope of this study.

**Table 2:** Statistically significant linear trends in the coefficients and constants in the PPRs.

Month	b1			b2			b3			D		
	Halls Gap	Swan Hill	Birchip	Halls Gap	Swan Hill	Birchip	Halls Gap	Swan Hill	Birchip	Halls Gap	Swan Hill	Birchip
January	Y^a^	Y	Y	N^b^	N	N	Y	Y	Y	Y	Y	Y
February	Y	Y	Y	N	N	Y	Y	Y	Y	N	Y	Y
March	Y	Y	N	Y	Y	Y	Y	Y	Y	Y	Y	Y
April	Y	Y	Y	N	N	N	N	N	Y	Y	Y	Y
May	Y	N	N	Y	Y	Y	Y	Y	Y	Y	N	Y
June	N	N	Y	N	Y	Y	Y	Y	N	Y	Y	Y
July	Y	Y	Y	Y	N	N	Y	Y	Y	Y	Y	Y
August	Y	Y	N	Y	Y	Y	Y	Y	Y	Y	Y	Y
September	N	Y	N	Y	Y	Y	Y	Y	Y	N	Y	N
October	Y	Y	Y	Y	Y	Y	Y	Y	Y	Y	Y	Y
November	N	Y	Y	Y	Y	Y	N	N	Y	Y	Y	N
December	Y	Y	Y	Y	Y	Y	Y	Y	Y	Y	Y	Y

^a^statistically significant linear trend in the coefficient/constant at 95% confidence level (p < 0.05).

^b^linear trend in the coefficient/constant statistically not significant at 95% confidence level

### Relationships between PPRs in SDM_1_ and statistics of reanalysis data

The changes in the statistics of global climate over time are regarded as indications of climate change. Hence, it was realised that relating the changes in the PPRs in SDM_1(MLR)_ and the statistics of the best potential predictors over time is a potential way to determine the influence of non-stationarities in the climate on the PPRs over time. For determining the relationships between the PPRs in SDM_1(MLR)_ and the statistics of the best potential predictors, initially, for each calendar month, for each 1-year movement of the 1^st^ window, the mean, the standard deviation and the 25^th^, 50^th^, 75^th^, 95^th^ percentiles of the NCEP/NCAR reanalysis data of the three best potential predictors were computed, for each station. In order to achieve this, 42 PPRs for each calendar month were considered for each station. The 1-year movement of the 1^st^ window yielded the time series of statistics of the reanalysis data of the three best potential predictors. By computing the correlations between each of these statistics and each coefficient/constant in the PPRs, the most influential statistic on each coefficient and the constant was determined. Table 3 shows the most influential statistic of the three best potential predictors on each coefficient and the constant in PPRs in SDM_1(MLR)_ for each calendar month, including their correlation coefficients (CC) for observation station at Halls Gap as an example. It was seen that the majority of the correlations were strong and statistically significant at the 95% confidence level for all stations.

**Table 3:** Most influential statistic of three best potential predictor on coefficients and constant in PPRs for observation station at Halls Gap.

Month	*b*_*1*_	*b*_*2*_	*b*_*3*_	D
January	25^th^ percentile of surface precipitation rate {(**4,4**)} (CC^b^ = ***-0*.*85***)	Standard deviation of 1000 hPa^a^ specific humidity {(**3,3**)} (CC = ***0*.*46***)	Average of 850 hPa meridional wind {(6,3)} (CC = *0*.*38*)	Average of surface precipitation rate {(**4,4**)} (CC = ***0*.*72***)
February	95^th^ percentile of surface precipitation rate {(**4,4**)} (CC = *0*.*32*)	Standard deviation of 1000 hPa relative humidity {(**4,3**)} (CC = ***0*.*52***)	Standard deviation of 850 hPa relative humidity {(3,1)} (CC = ***0*.*91***)	75^th^ percentile of 850 hPa relative humidity {(3,1)} (CC = ***0*.*76***)
March	Average of surface precipitation rate {(**3,4**)} (CC = ***0*.*94***)	Standard deviation of 850 hPa relative humidity {(2,3)} (CC = ***-0*.*53***)	25^th^ percentile of 1000 hPa specific humidity {(**4,4**)} (CC = ***-0*.*78***)	Average of 850 hPa relative humidity {(2,3)} (CC = ***0*.*82***)
April	95^th^ percentile of 850 hPa relative humidity {(**4,4**)} (CC = ***0*.*77***)	95^th^ percentile of 850 hPa geopotential height {(1,3)} (CC = ***0*.*42***)	50^th^ percentile of surface precipitation rate {(2,4)} (CC = ***-0*.*56***)	25^th^ percentile of 850 hPa geopotential height {(1,3)} (CC = ***0*.*92***)
May	25^th^ percentile of surface precipitation rate {(**4,4**)} (CC = *-0*.*34*)	50^th^ percentile of mean sea level pressure {(**4,4**)} (CC = ***-0*.*82***)	25^th^ percentile of 1000 hPa geopotential height {(**5,4**)} (CC = ***0*.*87***)	Average of mean sea level pressure {(**4,4**)} (CC = ***-0*.*89***)
June	95^th^ percentile of surface precipitation rate {(**3,4**)} (CC = *-0*.*22*)	95^th^ percentile of mean sea level pressure {(**3,5**)} (CC = ***-0*.*50***)	Standard deviation of 850 hPa zonal wind {(4,2)} (CC = ***0*.*67***)	Average of surface precipitation rate {(**3,4**)} (CC = ***0*.*97***)
July	Standard deviation of 850 hPa zonal wind {(4,1)} (CC = ***-0*.*72***)	Average of 850 hPa geopotential height {(**3,4**)} (CC = ***-0*.*73***)	Average of mean sea level pressure {(**4,4**)} (CC = ***0*.*77***)	95^th^ percentile of mean sea level pressure {(**4,4**)} (CC = ***-0*.*71***)
August	25^th^ percentile of surface precipitation rate {(**3,4**)} (CC = *0*.*38*)	95^th^ percentile of 850 hPa geopotential height {(4,6)} (CC = -0.46)	95^th^ percentile of mean sea level pressure {(5,6)} (CC = *0*.*39*)	25^th^ percentile of surface precipitation rate {(**3,4**)} (CC = ***0*.*52***)
September	25^th^ percentile of surface precipitation rate {(**3,4**)} (CC = *-0*.*35*)	50^th^ percentile of 850 hPa relative humidity {(**3,3**)} (CC = ***0*.*70***)	75^th^ percentile of 700 hPa relative humidity {(**3,3**)} (CC = ***-0*.*66***)	Average of surface precipitation rate {(**3,4**)} (CC = ***0*.*68***)
October	Standard deviation of surface precipitation rate {(**3,4**)} (CC = ***0*.*81***)	95^th^ percentile of 850 hPa relative humidity {(**3,4**)} (CC = ***0*.*63***)	Standard deviation of 700 hPa geopotential height {(2,2)} (CC = ***0*.*48***)	Average of 700 hPa geopotential height {(2,2)} (CC = ***-0*.*77***)
November	25^th^ percentile of 850 hPa relative humidity {(**3,3**)} (CC = *0*.*28*)	95^th^ percentile of surface precipitation rate {(**3,4**)} (CC = ***0*.*74***)	50^th^ percentile of 1000 hPa relative humidity {(**3,3**)} (CC = ***0*.*48***)	Standard deviation of 1000 hPa relative humidity {(**3,3**)} (CC = ***-0*.*66***)
December	95^th^ percentile of surface precipitation rate {(**4,4**)} (CC = ***0*.*82***)	Standard deviation of 850 hPa relative humidity {(2,3)} (CC = ***0*.*71***)	50^th^ percentile of 850 hPa specific humidity {(6,6)} (CC = ***-0*.*56***)	95^th^ percentile of surface precipitation rate {(**4,4**)} (CC = ***0*.*92***)

^a^atmospheric pressure in hectopascal.

^b^Correlation coefficient.

Bold text refer to the 9 closest grid points {(**3,3**),(**3,4**),(**3,5**),(**4,3**),(**4,4**),(**4,5**),(**5,3**),(**5,4**),(**5,5**)} to the site at Halls Gap post office. Bold italicised text refers to correlations statistically significant at the 95% confidence level.

### Modification of PPRs in SDM_1_ according to statistics of GCM outputs

**Fig 5:**
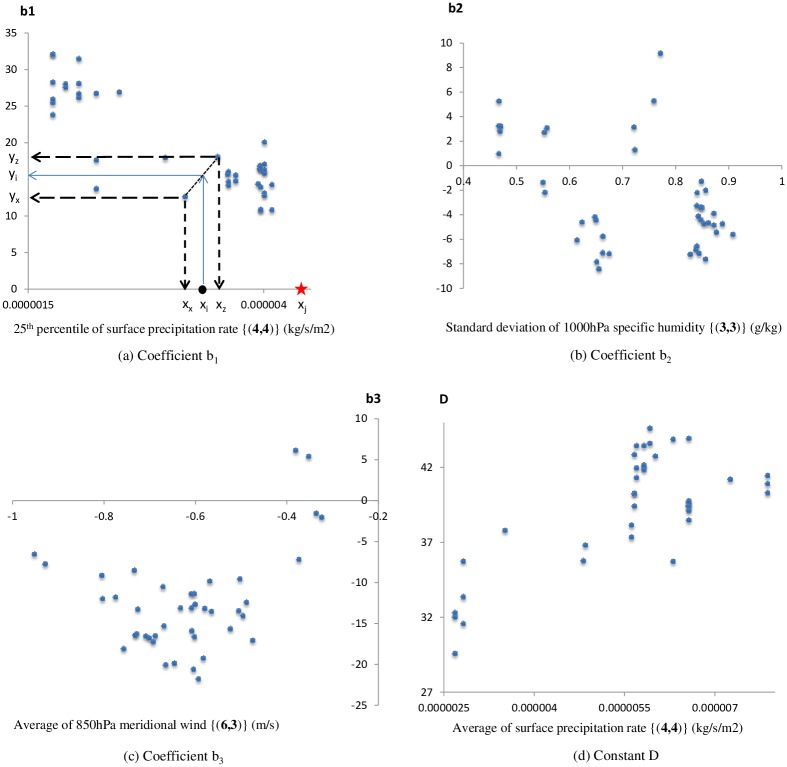
Scatter between coefficients/constant in PPRs and the most influential statistics of the three best potential predictors for January for Halls Gap station.

The next step of the modelling process was to modify the coefficients/constant in the PPRs of SDM_1(MLR)_ developed with the NCEP/NCAR reanalysis outputs according to the statistics of 20C3M outputs of HadCM3 GCM. For this purpose, initially, the variations of each coefficient and the constant of the PPRs in SDM_1(MLR)_ and the most influential statistic of the three best potential predictors were visualized using scatter plots. Fig 5 shows the scatter plots between the coefficients/constant in PPRs and the most influential statistic of the three best potential predictor for January as an example (scatter plots for other calendar months not shown) for the precipitation station at Halls Gap.

The statistics of the three best potential predictors pertaining to the past climate were then computed for 20-year time slices: 1950–1969 and 1970–1989 and another 10-year time slice: 1990–1999, using the 20C3M outputs of HadCM3. The 20C3M data for HadCM3 are only available till the end of the 20^th^ century. Therefore, the last time slice was limited to 10 years. Then using the scatter plots (e.g. Fig 5 for January at Halls Gap station), the values of the constant/coefficients corresponding to each value of the most influential statistic computed from the 20C3M outputs of HadCM3 were determined for the periods 1950–1969, 1970–1989 and 1990–1999, using linear interpolation as described in the next few paragraphs. In other words, the 42 x 12 PPRs derived using the NCEP/NCAR reanalysis data were used with the 20C3M outputs of HadCM3 for the derivation of 3 x 12 new PPRs (i.e. one PPR for each calendar month per time slice per station). It should be noted that the non-stationary downscaling models developed in this investigation are quasi-models, as the PPRs determined using the 20C3M outputs of HadCM3 for each period were stationary throughout that period.

As a demonstration for the application of linear interpolation to derive new values for the coefficients/constant in the PPRs of SDM_1(MLR)_, the following example is considered. In January, the 25^th^ percentile of surface precipitation rate at grid location {(4,4)} was the most influential statistic (correlation coefficient = -0.85) on the first coefficient (*b*_*1*_) of the MLR based PPR for Halls Gap station (see Table 3). As shown in Fig 5a, let the 25^th^ percentile of the surface precipitation rate at grid location {(4,4)} computed from the 20C3M outputs of HadCM3 for a certain period of interest (e.g. 1950–1969) be x_i_. To find the value of the first coefficient (*b*_*1*_) corresponding to x_i_, initially, the two points in the scatter (see Fig 5a) which referred to the values x_x_ and x_z_ of the 25^th^ percentile of the surface precipitation rate at grid location {(4,4)} closest to x_i_ on either sides of x_i_ are found. Then, by following the solid arrow line shown in Fig 5a, the value of the first coefficient (*b*_*1*_) pertaining to x_i_ is found as y_i_.

If the value of the most influential statistic of the potential predictor computed from the 20C3M outputs of HadCM3 were outside its range computed from NCEP/NACR reanalysis data, then the coefficient/constant determined using the NCEP/NACR reanalysis data was used without any modification. As an example, in Fig 5a, value of the 25^th^ percentile of the surface precipitation rate at grid location {(4,4)} x_j_ is outside the range of the scatter and there is no known value of coefficient *b*_*1*_ in the scatter pertaining to any x_k_ (>x_j_), hence interpolation is impossible. In such cases, extrapolation of the values of the coefficient/constant corresponding to the given value of the statistic pertaining to the past GCM data is seen as a solution. However, since extrapolation can introduce large errors to the estimated value of the coefficient/constant, it was not practised.

Like any other approach such as fitting a linear or a non-linear curve to the scatter between a constant/coefficient and a statistic of a predictor derived from reanalysis data, the linear interpolation between points in the scatter can also introduce uncertainties to the estimation of the values of the constant/coefficients pertaining to the values of the statistic of the predictor derived from the GCM data. In certain instances the uneven scatter (e.g. scatter with higher data density in certain regions than others) between the constant/coefficients and the statistic of predictor derived from reanalysis data was seen (e.g. Fig 5a). In the regions of the scatter where the density of data points is relatively high, the uncertainties introduced to the estimation of the values of the constant/coefficient can arise from the relative spread of the points, and in the regions of the scatter where the density of data points is low, uncertainties can arise due the absence of data points.

### Bias correction of GCM outputs and reproduction of observed precipitation

Once the coefficients and constant of the PPRs were determined corresponding to the statistics of the three best potential predictors pertaining to the 20C3M outputs of HadCM3 for the periods 1950–1969, 1970–1989 and 1990–1999, these new PPRs were used to reproduce the observations of precipitation for each calendar month, for each station using 20C3M outputs of HadCM3. Both reanalysis and GCM outputs contain bias, however, in general, GCM outputs tend to contain more bias than reanalysis outputs that are quality controlled and corrected against observations. The bias in the GCM outputs can propagate into the outputs of a downscaling model. Hence, it is an important task to correct the bias in the GCM outputs before any use. In this investigation, using the monthly bias-correction [29] the bias in the average and the standard deviation of 20C3M outputs of HadCM3 was corrected against the corresponding NCEP/NCAR reanalysis outputs for each calendar month.

In the application of the monthly bias-correction it is assumed that the bias in the variables over any period beyond the baseline period will remain the same as that of the baseline period. As the baseline period of the monthly bias-correction 1950–1969 was considered. Over the baseline period the monthly bias-correction was applied to the 20C3M outputs of HadCM3 by replacing their means and the standard deviations in each calendar month with the corresponding means and the standard deviations derived from the NCEP/NCAR reanalysis outputs pertaining to the same period. Beyond the baseline period (i.e. 1970–1989 and 1990–1999), the bias in the 20C3M outputs of HadCM3 were corrected by standardising these outputs with their means and standard deviations pertaining to the baseline period and rescaling with the corresponding means and standard deviations derived from the reanalysis outputs. Then the bias corrected 20C3M outputs of HadCM3 were used with those PPRs modified according to the statistics of 20C3M outputs of HadCM3 for the reproduction of observed precipitation for periods: 1950–1969, 1970–1989 and 1990–1999.

### Development of stationary downscaling models (SDM_2_)

For each station, two different types of conventional stationary downscaling models (SDM_2_) were developed: (1) a downscaling model based on MLR called SDM_2(MLR)_ and (2) a downscaling model based on GP called SDM_2(GP)_. The precipitation observations at the Halls Gap, Birchip and Swan Hill stations and the NCEP/NCAR reanalysis data of the three best potential predictors for the periods 1950–1969 and 1970–1989 were considered for the calibration and validation of SDM_2(MLR)_ and SDM_2(GP)_ respectively. For the calibration and validation of SDM_2(MLR)_ and SDM_2(GP)_, the above two periods were selected as it enabled the comparison of performance of SDM_2(MLR)_ and SDM_2(GP)_ with that of SDM_1(MLR)_ which was calibrated, validated and modified for the same periods. SDM_2(MLR)_ and SDM_2(GP)_ comprised of a set of 12 PPRs at a given station (each PPR pertaining to a specific calendar month).

In the development of SDM_2(MLR)_, for each calendar month, constants and coefficients of MLR-based PPRs were determined for the calibration period by minimising the sum of squared errors between the outputs of the SDM_2(MLR)_ and observations of precipitation. Thereafter, these PPRs were run with the reanalysis data of the three best potential predictors pertaining to the validation period.

For the development of SDM_2(GP)_ for each calendar month, for each observation station, the GP [30] technique was employed with the attributes shown in Table 4. The GP algorithm started with the random generation of a pool of downscaling models called an initial population using the observations of precipitation and the reanalysis data pertaining to the three best potential predictors for the calibration period. Then the fitness of each downscaling model in the initial population was assessed. Thereafter, the downscaling models in the initial population were selected for the mating pool based on their fitness. In the mating pool, genetic operations (e.g. crossover) were performed on downscaling models to generate a new population of downscaling models. Then, again the fitness of each downscaling model in the new population was assessed. In the above manner new generations of downscaling models were evolved iteratively until a predefined number of generations was met. Finally, the fittest downscaling model was identified for each calendar month for each station and run with the reanalysis data of potential predictors pertaining to the validation period.

**Table 4:** Attributes of Genetic Programming.

GP attribute	Description
Training and testing data %	Training 65% and testing 35%
Population size	500 members per generation
Program size/tree size	The maximum size of a member/maximum tree depth = 6
Terminals	Maximum number of inputs = 12, maximum number of constants in a model = 6
Mathematical Function set	+, -, x, ÷, √, x^2^, x^3^, sin, cos, e^x^, and ln
Initial population generation	Ramped half-half initialization
Fitness measure	Root mean square error (RMSE)
Selection criterion for mating pool	Fitness proportionate selection/roulette wheel selection
Mutation probability	0.05
Crossover probability	0.9
Replication probability	0.2
Stopping/termination criterion	Number of generations = 500

Once SDM_2(MLR)_ and SDM_2(GP)_ were calibrated and validated following the above procedure, they were used with the bias-corrected 20C3M outputs of HadCM3 as inputs for the reproduction of observed precipitation over the periods: 1950–1969, 1970–1989 and 1990–1999.

## Results

A statistical comparison of the performances of SDM_1(MLR)_, SDM_2(MLR)_ and SDM_2(GP)_ is presented in Tables 5, 6 and 7 for the three precipitation observation stations. The overall performance of each downscaling model was assessed with normalised mean square error (NMSE) in all three time slices: 1950–1969, 1970–1989 and 1990–1999. It should be noted that in the calculation of the normalised mean square error, the mean square error is normalised with the variance of the observed precipitation at a given station. This enabled the cross comparison of performance of downscaling models developed for stations pertaining to different climate regimes unlike the root mean square error or the mean square error which are sensitive to the order of magnitude of the predictand data.

**Table 5:** Performance comparison of SDM_1_ and SDM_2_ runs with NCEP/NCAR reanalysis outputs and 20C3M outputs of HadCM3 for Halls Gap station.

Time slice	Statistics	Observed	NCEP/NCAR outputs as inputs			HadCM3 outputs as inputs		
			SDM_1(MLR)_	SDM_2(MLR)_	SDM_2(GP)_	SDM_1(MLR)_	SDM_2(MLR)_	SDM_2(GP)_
1950–69	Average^a^	82.4	**82.5**	**82.5**	86.0	***80*.*4***	75.9	85.4
	Standard Deviation^b^	63	54.7	54.7	**58.9**	58.6	60.4	***62*.*8***
	5^th^ Percentile	8.6	**15.1**	**15.1**	19.0	***9*.*6***	10.2	11.2
	10^th^ Percentile	15.1	**22.9**	**22.9**	26.2	20.2	***15*.*1***	18.1
	25^th^ Percentile	35.4	**39.9**	**39.9**	41.5	***37*.*8***	32.6	39.8
	50^th^ Percentile	65.1	**70.0**	**70.0**	71.8	***65*.*5***	56.5	68.9
	75^th^ Percentile	118.5	**113.7**	**113.7**	123.6	112.1	***112*.*4***	128.3
	95^th^ Percentile	186.7	180.4	180.4	**191.2**	191.4	***184*.*7***	193.9
	NMSE^c^		**0.24**	**0.24**	0.30	1.33	1.25	***1*.*20***
1970–89	Average	81.2	**81.2**	82.5	86.9	84.8	86.3	***79*.*8***
	Standard Deviation	60.4	53.2	54.9	**59.5**	67.2	64.7	***60*.*5***
	5^th^ Percentile	9.3	**14.6**	15.4	14.9	7.9	7.1	***9*.*1***
	10^th^ Percentile	15.4	**22.2**	24.6	24.5	13.6	18.1	***15*.*4***
	25^th^ Percentile	34.7	**39.1**	42.3	43.0	33.0	39.6	***34*.*1***
	50^th^ Percentile	65.3	**69.0**	71.4	70.0	***64*.*9***	71.9	64.1
	75^th^ Percentile	121.1	116.0	109.5	**125.6**	128.7	***118*.*8***	114.1
	95^th^ Percentile	190.8	182.3	194.1	**191.0**	188.4	***192*.*3***	187.2
	NMSE		**0.22**	0.39	0.60	***1*.*33***	1.47	1.36
1990–99	Average	81.8	**80.2**	85.9	95.8	78.6	***84*.*7***	78.7
	Standard Deviation	64.4	54.5	57.5	**65.8**	58.8	60.2	***64*.*5***
	5^th^ Percentile	5.7	13.5	**7.1**	22.8	8.4	7.5	***5*.*2***
	10^th^ Percentile	17.6	**19.9**	23.1	32.7	***16*.*4***	13.5	11.6
	25^th^ Percentile	32.5	42.6	**41.7**	49.1	***33*.*9***	36.7	30.0
	50^th^ Percentile	62.3	**71.3**	76.2	73.3	***63*.*4***	74.3	58.7
	75^th^ Percentile	125.9	103.9	**109.3**	142.6	110.2	***124*.*2***	119.5
	95^th^ Percentile	218.4	189.3	205.4	**221.0**	***193*.*5***	***193*.*5***	189.1
	NMSE		**0.17**	0.35	0.41	***1*.*27***	1.29	1.34

^a^average of precipitation mm/month,

^b^standard deviation of precipitation mm/month,

^c^normalised mean square error.

Bold text refer to statistics of precipitation reproduced by downscaling models run with NCEP/NCAR reanalysis outputs which show the lowest bias in comparison to statistics of observed precipitation. Bold italicised text refer to statistics of precipitation reproduced by downscaling models run with 20C3M HadCM3 outputs which show the lowest bias in comparison to statistics of observed precipitation.

**Table 6:** Performance comparison of SDM_1_ and SDM_2_ runs with NCEP/NCAR reanalysis outputs and 20C3M outputs of HadCM3 for Birchip station.

Time slice	Statistics	Observed	NCEP/NCAR outputs as inputs			HadCM3 outputs as inputs		
			SDM_1(MLR)_	SDM_2(MLR)_	SDM_2(GP)_	SDM_1(MLR)_	SDM_2(MLR)_	SDM_2(GP)_
1950–69	Average^a^	31.9	**32.1**	**32.1**	32.9	***31*.*8***	32.5	34.9
	Standard Deviation^b^	24.8	**18.7**	**18.7**	18.2	28.0	***25*.*1***	29.0
	5^th^ Percentile	1.5	**5.2**	**5.2**	6.8	***1*.*2***	2.0	2.0
	10^th^ Percentile	4.4	**8.9**	**8.9**	12.0	3.8	***4*.*1***	3.6
	25^th^ Percentile	13.6	**19.0**	**19.0**	19.6	10.9	***13*.*8***	***13*.*8***
	50^th^ Percentile	27.6	**30.5**	**30.5**	30.9	24.5	***28*.*8***	28.9
	75^th^ Percentile	45.6	**43.6**	**43.6**	43.2	***45*.*6***	48.1	50.1
	95^th^ Percentile	81.0	62.3	62.3	**67.9**	83.5	74.4	***81*.*1***
	NMSE^c^		**0.40**	**0.40**	0.49	***1*.*97***	2.08	2.36
1970–89	Average	34.7	**34.1**	32.0	34.0	33.3	***34*.*0***	30.8
	Standard Deviation	29.3	20.9	18.6	**21.0**	25.8	***27*.*6***	24.4
	5^th^ Percentile	2.8	8.1	7.2	**4.9**	2.0	1.3	***2*.*1***
	10^th^ Percentile	4.9	**10.8**	11.4	10.9	5.2	4.3	***5*.*0***
	25^th^ Percentile	12.3	18.3	**17.2**	19.6	14.0	11.4	***11*.*8***
	50^th^ Percentile	28.1	**28.8**	29.9	31.0	27.5	***27*.*9***	24.9
	75^th^ Percentile	48.9	**47.8**	44.3	45.0	***47*.*6***	***47*.*6***	43.7
	95^th^ Percentile	83.9	66.4	65.3	**68.8**	81.0	***85*.*9***	80.5
	NMSE		**0.21**	0.59	0.43	1.70	1.81	***1*.*67***
1990–99	Average	31.3	**34.5**	34.7	36.0	34.4	***31*.*4***	33.1
	Standard Deviation	24.1	19.0	**20.9**	18.8	***25*.*2***	27.6	25.5
	5^th^ Percentile	1.2	8.2	**0.2**	9.7	***1*.*6***	2.1	***0*.*8***
	10^th^ Percentile	2.6	12.2	**11.2**	12.7	5.2	5.1	***4*.*2***
	25^th^ Percentile	13.3	20.2	**17.9**	21.5	14.7	***13*.*5***	12.7
	50^th^ Percentile	27.1	**31.5**	34.1	34.2	32.1	23.9	***27*.*6***
	75^th^ Percentile	46.7	46.3	**46.6**	48.4	48.3	41.9	***47*.*6***
	95^th^ Percentile	80.5	70.3	**72.5**	69.9	***80*.*3***	79.6	85.6
	NMSE		**0.24**	0.70	0.52	***1*.*84***	2.09	2.01

^a^average of precipitation mm/month,

^b^standard deviation of precipitation mm/month,

^c^normalised mean square error.

Bold text refer to statistics of precipitation reproduced by downscaling models run with NCEP/NCAR reanalysis outputs which show the lowest bias in comparison to statistics of observed precipitation. Bold italicised text refer to statistics of precipitation reproduced by downscaling models run with 20C3M HadCM3 outputs which show the lowest bias in comparison to statistics of observed precipitation.

**Table 7:** Performance comparison of SDM_1_ and SDM_2_ runs with NCEP/NCAR reanalysis outputs and 20C3M outputs of HadCM3 for Swan Hill station.

Time slice	Statistics	Observed	NCEP/NCAR outputs as inputs			HadCM3 outputs as inputs		
			SDM_1(MLR)_	SDM_2(MLR)_	SDM_2(GP)_	SDM_1(MLR)_	SDM_2(MLR)_	SDM_2(GP)_
1950–69	Average^a^	30.8	**30.8**	**30.8**	31.0	29.8	***31*.*2***	33.2
	Standard Deviation^b^	25.4	**19.7**	**19.7**	17.9	24.2	***25*.*4***	25.9
	5^th^ Percentile	2.1	**5.2**	**5.2**	6.1	***2*.*6***	1.4	1.5
	10^th^ Percentile	4.1	**8.3**	**8.3**	11.1	4.8	***3*.*8***	4.9
	25^th^ Percentile	10.9	**15.7**	**15.7**	19.0	11.3	***11*.*0***	12.6
	50^th^ Percentile	23.4	28.8	28.8	**28.1**	22.7	***23*.*9***	26.5
	75^th^ Percentile	44.5	**40.7**	**40.7**	38.7	***43*.*8***	45.4	48.1
	95^th^ Percentile	82.0	**70.3**	**70.3**	65.3	77.9	77.2	***84*.*6***
	NMSE^c^		**0.39**	0.39	0.46	***1*.*85***	2.04	1.95
1970–89	Average	32.7	**32.6**	30.9	**32.6**	***33*.*3***	32.0	29.1
	Standard Deviation	26.2	20.1	20.1	**20.2**	28.3	***27*.*0***	25.0
	5^th^ Percentile	1.5	8.9	5.7	**5.6**	***1*.*4***	1.8	2.0
	10^th^ Percentile	4.6	11.1	10.7	**9.6**	3.7	***3*.*8***	***3*.*8***
	25^th^ Percentile	13.4	17.9	**16.6**	17.3	10.8	***11*.*4***	11.0
	50^th^ Percentile	24.5	27.3	**27.0**	29.3	23.7	***24*.*1***	22.3
	75^th^ Percentile	48.9	**44.4**	41.1	44.3	***51*.*8***	45.5	40.9
	95^th^ Percentile	79.6	70.8	**75.5**	70.2	87.8	91.2	***77*.*4***
	NMSE		**0.21**	0.62	0.43	2.26	2.05	***1*.*88***
1990–99	Average	29.4	**32.9**	34.0	33.7	30.1	***29*.*9***	31.6
	Standard Deviation	25.6	19.3	**19.8**	18.9	23.3	24.0	***26*.*8***
	5^th^ Percentile	1.1	7.6	**6.9**	8.5	1.5	2.0	***1*.*4***
	10^th^ Percentile	3.8	12.6	12.9	**11.9**	4.0	6.0	***3*.*7***
	25^th^ Percentile	10.4	**18.3**	19.2	20.1	12.7	11.4	***10*.*2***
	50^th^ Percentile	22.1	**27.1**	29.7	29.5	26.2	21.3	***22*.*5***
	75^th^ Percentile	44.4	48.0	48.5	**47.1**	44.2	***44*.*5***	47.7
	95^th^ Percentile	75.7	70.4	67.5	**71.5**	***76*.*2***	71.1	76.4
	NMSE		**0.22**	0.64	0.44	***1*.*64***	1.93	2.10

^a^average of precipitation mm/month,

^b^standard deviation of precipitation mm/month,

^c^normalised mean square error.

Bold text refer to statistics of precipitation reproduced by downscaling models run with NCEP/NCAR reanalysis outputs which show the lowest bias in comparison to statistics of observed precipitation. Bold italicised text refer to statistics of precipitation reproduced by downscaling models run with 20C3M HadCM3 outputs which show the lowest bias in comparison to statistics of observed precipitation.

According to Tables 5, 6 and 7 it was seen that, when SDM_1(MLR)_ for the stations at Halls Gap, Birchip and Swan Hill were run with NCEP/NCAR reanalysis outputs of potential predictors, it displayed the lowest NMSE in all three time slices in comparison to NMSEs of SDM_2(MLR)_ and SDM_2(GP)_. This was due to the fact that unlike SDM_2(MLR)_ and SDM_2(GP)_ which were only calibrated for period 1950–1969, SDM_1(MLR)_ was calibrated using a 20-year moving window moved at a 1-year time step that included the periods: 1950–1969, 1970–1989 and 1990–1999.

As shown in Table 5 which refers to the Halls Gap station located in relatively wet climate, when SDM_1(MLR)_ was run with the 20C3M outputs of HadCM3, it displayed the smallest NMSE over periods 1970–1989 and 1990–1999 in comparison to that of SDM_2(MLR)_ and SDM_2(GP)_. Also, SDM_1(MLR)_ was able to better reproduce the 50^th^ percentile of observed precipitation than that by SDM_2(MLR)_ and SDM_2(GP)_ in all three time slices. However, SDM_2(MLR)_ was able to capture the 75^th^ and 95^th^ percentiles of observed precipitation better than those by SDM_1(MLR)_ and SDM_2(GP)_, in all three time slices when it was run with 20C3M outputs of HadCM3. Also, in all three time slices, the standard deviation of observed precipitation was better simulated by SDM_2(GP)_ than that by SDM_1(MLR)_ and SDM_2(MLR)_ with the 20C3M outputs of HadCM3.

According to Tables 6 and 7 which refer to the Birchip and Swan Hill stations located in intermediate and dry climate respectively, it was noted that, with the 20C3M outputs of HadCM3, SDM_1(MLR)_ was able to display the lowest NMSE over the periods 1950–1969 and 1990–1999. However, it was seen that with the 20C3M outputs of HadCM3 no downscaling model was able to outperform the others in better reproducing any of the statistics (e.g. percentiles) of observed precipitation consistently in all three time slices at Birchip or Swan Hill stations.

## Discussion

When run with the 20C3M outputs of HadCM3 (past GCM outputs) it was seen that no downscaling approach (stationary or non-stationary) was able to consistently outperform the other approaches in all three time slices at any of the three stations, in terms of normalised mean square error (NMSE). However, the downscaling models based on the non-stationary approach (SDM_1(MLR)_) were able to display the lowest NMSE at all stations in two of the three time slices with the 20C3M outputs of HadCM3. This indicated that the downscaling models based on the non-stationary approach were able to produce more accurate simulations of observed precipitation more often than linear and non-linear downscaling models based on the conventional stationary approach. For all stationary and non-stationary downscaling models when run with the 20C3M outputs of HadCM3 it was seen that the NMSE was relatively higher for the stations located in the dry and intermediate climate in comparison to that for the station located in the wet climate. This hinted that, irrespective of the downscaling approach, higher degree of error is associated with the simulations produced by the downscaling models pertaining to relatively dry climate.

In the application of the SDM_1(MLR)_ for future climate, the constants/coefficients of PPRs developed using the reanalysis data of predictors and the observations of precipitation should be updated according to the future climate simulated by the GCM for deducing new non-stationary PPRs pertaining to the future. For that purpose, scatter between the statistics of reanalysis data of predictors and constants/coefficients of PPRs for the past climate and statistics of data of predictors simulated by the GCM for the future are used. In such instances, there is a likelihood that the statistics of predictors derived from GCM data for the future lie outside the range of statistics of predictors derived from the past reanalysis data. This issue can make the model less non-stationary, as extrapolation of a value of a constant/coefficient outside the range of reanalysis data is discouraged. Such likelihood, will be higher in the distant future and smaller in the near future and may make the non-stationary downscaling model developed for the distant future to be more stationary rather than non-stationary. However, with time, the continuous updating of the scatter between the statistics of the predictors derived from reanalysis data which is expanding over time and the constants/coefficients in the PPRs, will minimize of likelihood of having the statistics of the predictors derived from GCM data for future to lie outside the range of the reanalysis data. Alternatively, a non-linear regression technique such as Genetic Programming can be used to develop a relationship between the values of a statistic of a predictor derived from the reanalysis data and the values of a coefficient/constant in the PPR of interest. Then this non-linear regression relationship can be used with the values of the statistic of the predictor derived from a GCM database which lie outside the range of past reanalysis data to determine the corresponding values of the coefficient/constant in the PPR. This regression based approach can even be used in conjunction with the already proposed continuous updating of the scatter between the statistics of the predictors derived from reanalysis data and the constants/coefficients in the PPRs.
